# The Automatic Methods Group *Newsletter*

**DOI:** 10.1155/S1463924697000126

**Published:** 1997


Journal of Automatic Chemistry, Vol. 19, No. 4 (July–August 1997) pp. 133–139

**AUTOMATIC METHODS GROUP**

Analytical Division
ROYAL SOCIETY OF CHEMISTRY

### The Automatic Methods Group *Newsletter*

The AMG Newsletter is published as part of and in collaboration with the *Journal of Automatic Chemistry*. Further information on the *Journal of Automatic Chemistry* can be obtained from Taylor & Francis Ltd, Rankine Road, Basingstoke, Hampshire RG24 8PR or e-mail:info@tandf.co.uk; worldwide web home page: http:||tandf.co.uk.

### Meeting Reports

### Field test kits—Analysis outside the laboratory

A joint meeting of the Automatic Methods Group of the Analytical Division, The Royal Society of Chemistry and The Association of Clinical Biochemists was held on Thursday 28th November 1996 at The Geological Society, Burlington House, Piccadilly, London W1.

This joint topic meeting between the RSC's Automatic Methods Group and the Association of Clinical Biochemists explored some of the problems associated with staff training, quality assurance and data integration when analysis is done outside the confines of the traditional laboratories.

The programme was grouped into two complementary half-day sessions—broadly clinical in the morning and industrial/analytical in the afternoon.

The morning session began with Prof. Vadgama's update on electrochemical sensors. His Manchester group had studied in-dwelling tissue electrodes for some years, as a possible alternative to venepuncture, but *in situ* poisoning of the electrodes had always been a problem. A newly-developed continous-flow technique, however, significantly improved both electrode sensitivity and effective duration.

Dr. Ken Paterson from Glasgow then presented the results of the British Diabetic Association's working party, outlining where and when it is appropriate to measure glucose locally.

Some analyses have been successfully improved and simplified for unskilled users. One such is $\beta$-hCG, the core of commercial pregnancy tests. Keith May of Unipath presented both the technical background to the assay, and the developments necessary to simplify a complex immunoassay to produce an easy-to-use method and display a clear-cut answer.

Finally, Prof. Packard from Glasgow Royal Infirmary discussed the UK's nationwide cholesterol improvement and monitoring program, in the context of advice from Health Authorities in the classification and treatment of coronary risk.

The afternoon session continued the quality theme with a discussion of the Laboratory of the Government Chemist's VAM initiative on validation of field test kits from Dr. Bethan Chapman of LGC.

Often the only practical way of measuring gaseous environmental pollutants in the workplace involves on-site monitoring. Dr. Schirk from Draegerwerk AG, Luebeck presented his company's approaches to diffusive monitoring for formaldehyde and ozone testing using selective enzyme-linked or direct colour reactions incorporated into a badge system.

And finally, the shake-up in the water industry in England and Wales has vested responsibility in the National Rivers Authority to monitor the continued cleanliness of the rivers and ensure that industrial effluent and agricultural run-off conform to the various statutes on maximum permitted levels of contaminants. Dr. John Cope, previously of Severn–Trent Water, now an independent consultant, presented details of riverside testing for aqueous pollution.

The meeting attracted a small but enthusiastic audience, whose questionnaire response indicated a positive view of the quality of the speakers and topic. The abstracts presented follow.

**Alan S. McLelland**

*Biochemistry, Glasgow Royal Infirmary, Glasgow, UK*

0142-0453/97 $12·00 © 1997 Taylor & Francis Ltd

### Abstracts of papers presented

### Electrochemical Sensor Systems—Biochemistry without venepuncture

**G. P. Rigby, S. Ahmed and P. Vadgama**

*Department of Medicine, University of Manchester, Hope Hospital, Salford M6 8HD, UK*

Access to the blood compartment remains the mainstay of clinical biochemistry testing, not least in the context of diabetes. Though home monitoring has been possible through the use of capillary blood sampling, followed by simplified measurement with either electrochemical or reflectance based dipsticks, such an approach is only feasible for intermittent measurement. The high variability of blood glucose in diabetes, coupled with the need to tailor insulin administration for better control, would seem to warrant real-time continous monitoring. It is now generally accepted that the extravascular tissue compartment is the safest access route to glucose monitoring, avoiding the dangers of thromboembolism and disseminated infection.

The needle enzyme electrode exploiting a relatively stable enzyme (glucose oxidase) with a self-contained two electrode (Pt, stainless steel) electrochemical cell has provided an ideal structure for tissue implantation [1]. However, the tissue matrix has proved to be a much more difficult environment in which to achieve meaningful quantitative measurement. There is a multiplicity of microenvironments which have a largely uncharted biochemical relationship with the blood compartment. Moreover, analyte mass transport to an electrode sensing surface is limited by constituent biopolymers. There is also an inevitable foreign body tissue response that distorts the local matrix further.

We have developed an open microflow system that exploits the negative hydrostatic pressure of tissue for pumpless delivery of isotonic fluid around the implant site. This hydrates the tissue, controls local 'solvent' conditions and allows pharmacological manipulation of any tissue response. The result is that for the first time, *in vivo* calibration is avoided, blood: tissue levels are equivalent and stabilization of electrodes is achieved in minutes [2]. The approach shows promise for *in vivo* electrochemical sensors generally.

Other tissue access techniques to be described are (1) microdialysis, a universal sampling technique [3] usable with *ex vivo* sensors, which may eventually prove suitable for ambulatory monitoring (2) suction effusion, which attempts to collect subcutaneous tissue transudate for *in vitro* measurement and (3) heated skin electrodes which allow transcutaneous respiratory gas monitoring.

### References

1. Christie, I. M. and Vadgama, P. M. 5 (1994) 131.
2. Rigby, G. P., Crump, P. W. and Vadgama, P., *Analyst*, **121** (1996) 871.
3. Benveniste, H. and Huttemeir, P. C., *Prog. Neurobiol.* **35** (1990) 195.

### Acknowledgement

Grateful thanks are given to the British Diabetic Association for generous support to GPR.

### The science behind home pregnancy testing

**Dr. Keith May**

*Unipath Ltd*

The first home pregnancy test kits were sold in the early 1970s and were based on latex or red blood cell agglutination. The tests were basically repackaged laboratory tests with test tubes, droppers, and were difficult to interpret and not widely used. The development of monoclonal antibody technology with the ability to produce unlimited quantities of antibody with defined specificity fuelled technology developments which led to improvements in the speed, simplicity and reliability of home pregnancy kits. The introduction of Clearblue in 1985 represented a shift from a technology focus to a clear identification of the consumer needs. The product started to address what consumers wanted by addressing (1) urine collection (2) no test tubes or droppers (3) easy to read results and (4) faster results (30 min). This was superseded in 1988 by the introduction of Clearblue one step which was the first one-step pregnancy test and provided a result in less than 5 min. The product has been improved since the introduction of a 1 min version which can detect 50 mIU of hCG (the level observed on or before the first day of the missed period) has recently been introduced. The product is able to provide the combination of speed and sensitivity through the use of high concentrations of specific monoclonal antibodies in an immunochromatographic format. The use of monoclonal antibodies has allowed the routine and repeated production of product with controlled specificity. By careful selection of reagent combinations it has been possible to develop tests which detect all isoforms of hCG, e.g. nicked deglycosylated, etc. In addition, the standardization of the product with well-defined and characterized reference preparations free of contamination with free subunits have allowed the routine production of tests with controlled sensitivity. As the product is produced according to well-defined manufacturing processes, and is subject to rigorous quality assurance and quality control procedures, its performance batch to batch is assured. Clearblue one step has been extensively tested in both laboratory and home settings, and has been found to be 99% + accurate. Because Clearblue one step is extremely simple to perform, the results obtained by the consumer are very accurate and similar to those obtained in the laboratory.

### National proficiency testing of cholesterol methods and users

**Chris J. Packard**

*Institute of Biochemistry, Glasgow Royal Infirmary, University NHS Trust, Castle Street, Glasgow G4 OSF, UK*

Proficiency testing of cholesterol assays is a critical component of the delivery of preventive cardiology. All who perform both near-patient analyses or definitive laboratory determinations should be aware of the potential bias and imprecision in their assays. Traditional approaches to quality control of serum cholesterol measurement are fraught with difficulties since most rely on artificial materials, usually animal based, they are lyophilized for the purposes of preservation and transport. Enzymatic cholesterol assays, particularly those which involve dry reagent technology are susceptible to matrix effects so that the detection of bias in normal QA scemes is unreliable. Traceability to an accuracy base can only be performed, at the moment, with authentic human sera which are fresh or frozen immediately on harvesting. A mechanism for assuring the accuracy of cholesterol determination in lipid research and epidemiological laboratories has been in place for a number of years. Organized by the Center for Disease Control in Atlanta, GA, an international network of about 12 laboratories circulate materials which are assayed by the reference (Abell-Kendal) method. The network maintains the precision of its AK methods to within 2% CV and a bias of <1%. These laboratories then offer standardization to client clinical chemistry laboratories, manufacturers or quality control schemes in their local region. Recently, HDL cholesterol and serum triglyceride have been added as analytes which can now be traced through this system.

A quality control scheme for the proficiency testing of the Reflotron desk-top dry chemistry analyser, has been operated by Glasgow Royal Infirmary, Department of Biochemistry, for a number of years. A companion scheme has been offered for NPT devices by the Wolfson Laboratory NEQAS organizers over a similar time scale. In both instances sera with target values assigned by the Network Laboratory at GRI are distributed to sites involved in screening in general practice, clinical trials or hospital clinics. Precision and accuracy limits are set at <5% CV and <5% bias and the vast majority of operators achieve this performance on a regular basis. It is unlikely that the more stringent criteria of <3% bias and imprecision will be met in the field testing environment and this limits the usefulness of these devices. They can be used for patient classification and in coronary screening clinics, but not for therapeutic decisions.

### Importance of using valid test kits for analysis

**Dr. Bethan R. Chapman**
*Laboratory of the Government Chemist*

The Laboratory of the Government Chemist has carried out a number of validation studies on analytical test kits, covering the food, environment and forensic sectors. This work was supported under contract with the Department of Trade and Industry as part of the National Measurement System Valid Analytical Measurement (VAM) programme. One of the projects under this programme is 'Quality assurance of test kits' and two of the key aspects of this project are to explore the performance of existing test kits and develop guidance on methodology for test-kit assessment.

This presentation will aim to convey the importance of using valid test kits for your analysis. A consideration of factors affecting the validity of test kits results, such as sensitivity, matrix effects, quality control and repeatability, will be given along with an outline of methods suitable for testing these factors. In addition, examples will be given from a range of test kits as to what can go wrong if a test kit is not performing to the specified criteria. Finally a brief introduction will be given to a number of schemes already available for certifying test kits, and how end users can utilize this information.

The aim of this talk is to demonstrate the importance of using valid test kits and to give end users the information they need in order to identify test kits that are fit for their purposes.

### Spot testing and diffusive monitors for gaseous emissions

**Oliver Schirk**

*Drägerwerk AG Lübeck, Germany*

1. Introduction

Direct reading tubes and active sampling systems are used for work place and indoor-air measurement. Both systems usually require a pump. A defined volume of air has to be pumped through the direct reading tube or the sampling system. Diffusive monitors offer the advantage of working without any additional equipment. The user does not need any pump and thus a pump calibration step is not necessary. Two different direct reading diffusive monitors are introduced in this paper.

2. Formaldehyde measurement using a diffusive monitor

The diffusive monitor for formaldehyde measurement (Bio-Check F) is a badge system. Bio-Check F is a biosensor with an optical transducer. It does not generate an electrical signal like other biosensors do, i.e. evaluation is performed with the naked eye and does not require any additional equipment. The enzymes and reagents required for the measuring process are arranged in a badge system which contains a sintered glass rod and an ampoule filled with an activation solution.

The ampoule is broken by pressing the start button whereby the indicator-layer (top of the sintered glass rod) of the Bio-Check F is moistened, which in turn activates the enzymes (formaldehyde dehydrogenase and diaphorase). Should formaldehyde be present, the colour of the diffusion layer changes from white to pink or even red. The formaldehyde concentration can be established after a measurement time of 2 h by comparing the discoloration with an attached colour code and using an evaluation table.

The enzymatic measurement system of Bio-Check F is extremely selective so that in addition to formaldehyde, acetaldehyde is indicated with only approximately 50 times less sensitivity. Bio-Check F is usable for a single formaldehyde measurement.

3. Ozone measurement using a diffusive monitor

Measurement of ozone can be done with a badge measuring system containing test strips and a colour coded for evaluation (Bio-Check Ozone). The measurement is performed by inserting an ozone-sensitive test strip into a badge holder. The function principle of Bio-Check Ozone is the oxidation of potassium iodine by ozone. After a duration of 20 min for indoor-air measurement or 10 min for outdoor measurements, the ozone concentration can be evaluated by comparing the discoloration of the test strip with the colour code printed on the badge holder.

The test strips of Bio-Check Ozone are impregnated in a special way so that nitrogen oxides are indicated with approximately 16-times less sensitivity. Hydrocarbons and sulphur dioxide are not indicated. Chlorine is indicated with similar sensitivity, but the shade is reddish and quickly becomes dark after the measurement. If chlorine occurs simultaneously with ozone, the measured value will be too high.

### Petroanalysis '97

### A joint meeting of the Automatic Methods Group of the Royal Society of Chemistry and the Institute of Petroleum held on Wednesday 21 May 1997 at the BP Oil Technology Centre, Sunbury-on-Thames, Middlesex, UK

The programme for Petroanalysis '97 included the following presentations:

*Morning session: Chairman: Harry Read (BP Oil Technology Centre, Sunbury on Thames, UK):*

Petroanalysis, where are we now?
*Chris Bartlett (DRA, Farnborough, UK).*

Automated determination of base and acid numbers *Malcolm Fox (Department of Chemistry, De Montfort University, Leicester, UK).*

Microwave spectrometry applied to the petrochemical industries *Fred Alder (DIAS, UMIST, Manchester, UK).*

Recent Developments in Gasoline Analysis using IR Spectroscopy *Greg Brown (PetroSpec Inc., Tuftin, California, USA).*

*Afternoon session: Chairman: Bob Hooks (Shell Research and Technology Centre, Thornton, UK):*

Test houses for the next millennium *Paul Slater (Fuchs Lubricants, Hanley, UK).*

Inter-centre precision monitoring schemes *Harry Read (BP Oil Technology Centre, Sunbury on Thames, UK).*

Surface studies of oil seal degradation *Rod Davies and G. Smith (Shell International Oil Products Ltd, Thornton, UK).*

Elemental analysis by X-ray fluorescence *Ken Field (Oxford Instruments, Abingdon, UK).*

Automated instrumentation for on-ship monitoring *Chris Leigh-Jones (Kittewake Ltd, Littlehampton, UK).*

### Petroanalysis, where are we now?

**Chris Bartlett**
*Defence Evaluation and Research Agency, Farnborough, UK*

The paper began with the question 'Petroanalysis, where did we come from?' and some personal reminiscences of nearly 40 years of petroleum analysis. This has been a period of major change, with the widespread application of instrumental and automated methods, the consequent reduction in staff numbers in analytical laboratories, and the gradual improvement in precision arising from the introduction of laboratory accreditation schemes.

The question 'Where are we now?' was posed in the light of the developments over the last 40 years with some consideration of the role and ethics of the petroleum analyst, and of the training of the next generation of analysts.

Finally, the question 'Where are we going?' was asked with particular relevance to the continued development of efficient and automated techniques both in the laboratory and in the operating environment, and to the continuing role of the Institute of Petroleum (IP) in the field of international standardization.

### Automated determination of base and acid numbers

**D. A. Armitage, Malcolm F. Fox and S. M. Pickering**
*De Montfort University, Leicester, UK*

Determinations of base and acid numbers for fresh and used lubricating oils are difficult and relatively imprecise by potentiometric titration and unsuitable for automation. New developments in procedures, mainly due to conductimetric titrations, have given a new impetus to automating base and acid number determinations, with greater precision and significantly smaller volumes of sample.

Two automated methods were described for the base number determination of lubricating oil samples, one for a multisampler, continuous operation device as part of a condition monitoring system which is completely automated after sample loading with a throughout of 10–15 samples/h. The second system is for intermittent use with manual introduction via an electronic top-pan balance followed by an automated titration.

Both systems have been extensively and successfully tested with large numbers of determinations drawn from fresh, used and extensively degraded samples, from a wide range of original lubricant formulations and from automotive petrol and diesel, marine diesel and concentrated additives. Results are significantly within the IP requirements for reproducibility and repeatability. The previous problems of determining base number involved the electrode system. The advantages of the automated conductimetric titration system were assessed and solutions to its development problems described.

Acid number determination was developed as a simple, sequential extension of the base number determination of the same sample in the same reaction vessel. The base number determination has been incorporated into a multiparameter diesel engine condition monitoring system and further incorporated into a water and particulate content analyser with preproduction models built for evaluation.

### Microwave spectrometry applied to the petrochemical industries

**J. F. Alder**
*UMIST, Manchester, UK*

**and J. G. Baker**
*University of Manchester, Manchester, UK*

Microwave (MW) and Millimetre Wavelength Spectrometry (MMWS) are powerful analytical methods for the determination of gaseous constituents in a wide range of atmospheres. Being sensitive to molecules possessing a dipole moment, and insensitive to gases including atmospheric constituents (with the notable exception of oxygen), and the low molecular weight hydrocarbons, these techniques are ideally suitable for determinations in flue gases and hydrocarbon atmospheres, as well as atmospheric air.

MW and MMWS have the particular feature of high specificity to individual molecular species, including positional and isotopic species, with part per million (ppm) sensitivities for the strong lines and 100 ppm sensitivities for the higher MW gases. The authors have already demonstrated the potential of MWS for water determination in hydrocarbon gases, and a range of molecules: NO, $SO_2$, HCHO, acrolein, acrylonitrile, ammonia, OCS, etc.

A particular MMWS study of the determination of oxygen at 60 GHz in air, nitrogen and carbon dioxide atmospheres over the range zero–100% oxygen in the last two, has shown that the atmospheric composition has no noticeable effect upon the response curve for oxygen, as indeed is predicted by the theory of microwave spectrometry; likewise for the determination of water at 22 GHz in air and LPG. This shows the way towards the application of MMWS to a wide range of petroleum industry related determinations associated with feedback and emission control analysis. The paper presented an overview of the theory and practice of MMWS for real world analysis.

### Recent developments in gasoline analysis using IR spectroscopy

**Greg Brown**
*PetroSpec Inc., Tuftin, California, USA*

Optical measurements in the infrared (IR) and Near Infrared (NIR) are proven technologies in many refineries, pipelines, terminals and custody transfer stations. Scientists have employed these technologies for years, but the technical expertise, sample preparation requirements, or robustness of the analysers limited application of the technology outside the laboratory walls.

Recent technological advances enable many complex tedious laboratory analyser measurements to be made now with robust portable IR and NIR analysers in front line operations by non-technical personnel with a high degree of reliability and accuracy. Further, many of these technologies are now being transferred to terminals, pipelines, refinery mini-labs, to surveillance and enforcement teams and are on-line at refineries.

Automotive and engine manufacturers around the world are discussing with refiners, the need for similar fuel recipes, so that power trains built in Detriot, the UK and Japan will work in Brazil, Europe and China to the same optimum performance. World-wide, governments consider pollution and enact emissions regulations. Many of the components, constituents, and regulatory requirements can be measured in the IR and NIR today. Rugged, easy to use, portable IR analysers, and industrially hardened on-line process analysers offer new ways to improve yields, speed processes, and increase economic returns.

Measurements of octane number, total aromatics, benzene, and oxygenates, are often routine with these analysers. Olefins, saturates, RVP, distillation points, and percentage of transmix can also be measured in the IR. The engine and automanufacturers are also driving a push for driveability index (DI). The driveability index is a combination of three weighted distillation points at T10, T50, and T90. Often DI is a better indication of an automobile fuel problem than octane number.

IR and NIR analysers can now be found from crude applications, through refinery unit operations, working on-line at the blender, along the pipeline, to the terminal, and in the hands of surveillance and inspection teams of the US EPA and IRS. IR, FTIR, and NIR suppliers offer equipment which speeds throughput, reduces reblends, mediates transfer disputes, and ensures product quality.

### Test houses in the next millennium

**P. M. Salter**
*Fuchs Lubricants, Hanley, UK*

At present, most lubricant companies throughout the world have laboratory facilities at their disposal. Fuchs Lubricants are no exception to this, particularly in the UK and an outline of these facilities was given to establish a datum for the discussion of future developments. Fuchs Lubricants UK has two sites which both have laboratory facilities: Hanley and Belper. The features of these laboratories are:

- *Hanley plant:* Quality Control, R&D—Industrial, customer service/analytical, CENT condition monitoring. These sections use ICP, GC, IR, FTIR, KV100, Karl Fischer, PQ, TAN, TBN and other test methods.
- *Belper plant:* Automotive R&D, petroleum jelly, quality control, customer service. These sections use XRF, GC, AA, IR, FTIR and other test methods.

Test houses should be working towards common goals during the next millennium. These should take the form of a standards committee to formulate new test methods and refine existing methods, and a quality standard for all laboratories to prescribe to instead of having to meet various standards set by various disciplines.

Integration is the key and this is being utilized in different diciplines, such as engineering, where mechanical and electrical organizations are merging to form a common committee. The cost reduction benefits of this are apparent and the 'strength in numbers' doctrine is to their advantage.

These are matters that should be dealt with in terms of test house to test house, but there are greater opportunities to be achieved between test house and customer. Customers, whether internal or external, require information fast and preferably by computer rather than a paper medium. Oil companies now supply condition monitoring information and lubricant survey information via floppy disk/modem links so that the customer has instantaneous access to their details. This is now demanded by the customer as a right, not a privilege, and we must recognize this fact.

Test houses need to be prepared for places like the internet, infranet and world-wide webs to share and export their knowledge and information and utilize neural networks for analytical capabilities. This should be coupled by universal standards that are achievable by everyone.

### Intercentre precision monitoring schemes

**Harry Read**
*BP Oil Technology Centre, Sunbury on Thames, UK*

With the increase in requirements for accreditation in oil industry laboratories, the use of some form of scheme which will allow the laboratory to check its analytical testing performance is essential. The various schemes available to oil industry laboratories were discussed, outlining the differences that exist between schemes run on a national basis, such as the ASTM crosscheck programme, and those run within multinational companies such as BP.

The major part of the presentation looked at the benefits of belonging to such a scheme, not only to check laboratory performance, but also the use of the monthly reference sample that may be used as an in-house 'secondary standard'. Details of how the BP ICPMS scheme is run were discussed, along with some examples of how the test data are statistically processed and graphically produced.

The use of such schemes to offer the benefits of enhancing staff training and allow the laboratory to demonstrate competency to both customers, internal and external, as well as external accreditation bodies was stressed.

### Surface studies of oil-seal degradation

**G. C. Smith* and R. E. Davies**
*Shell International Oil Products Ltd, Thornton, UK*

Fluoroelastomers are frequently used as engine oil seal materials. Under certain test conditions, specific fluoroelastomers may show degradation of mechanical properties. The authors have used a range of surface and structure-sensitive analytical techniques to investigate the physical and chemical changes associated with degradation of a variety of fluoroelastomer materials aged in simple oil/additive blends and in oil formulations equivalent to commercial blends. X-ray diffraction (XRD) revealed changes in the crystalline fillers present in the fluoroelastomers. X-ray photoelectron spectroscopy (XPS) showed details of the degradation of the polymer components and the degree of cross-linking. Scanning electron microscopy with electron probe micro-analysis (SEM/EPMA) gave information on structural and chemical changes and the depth of degradation. The interaction was found to proceed through amine catalysed post-curing of the constituent polymers. These reactions promote defluorination, embrittlement and cracking of the elastomers, with a consequent decline in tensile properties as fracture failure mechanisms dominate performance. Even in the most extreme case, degradation was found to be limited to depths of less than approximately 50 µm. Degradation was reduced in elastomers with a higher fluorine level, higher terpolymer content, and greater degree of cross-linking.

* Present address: Shell Research and Technology Centre, Amsterdam, The Netherlands.

### Elemental analysis by X-ray fluorescence

**Kenneth Field**
*Oxford Instruments, Abingdon, UK*

Over the past few years the variety of X-ray fluorescence analysers has increased leading to their wider use for both established and new applications. The first part of this paper gave an overview of the types of instrument now in use and covered simple energy dispersive, high resolution energy dispersive, sequential wavelength dispersive, simultaneous wavelength dispersive and the hybrid multi-dispersive X-ray fluorescent spectrometers.

Even if one only considers quality and process control analysis, there are still many areas of use for X-ray fluorescence that fall into four broad classes. These are routine determination of one or few elements, routine determination of five or more elements and routine analysis plus variety of non-routine tasks. Finally, there are new applications that demand new ways of working. A number of routine applications are described and generally there is a correlation between the complexity of the analytical task and the size of the instrument. However, there are some cases where the best solution is a wavelength dispersive instrument in a simple configuration.

Two new uses of high resolution (solid state detector) energy dispersive instruments were covered: these were the race track measurement of engine wear metals and the difficult problem of analysing liquid hazardous waste.

Two external influences were considered: the need to keep up with regulatory demands on analytical techniques, and the requirement to implement standard test methods. In particular, as limits for sulphur in fuels continue to drop there has been a continual chase between the performance of simple energy dispersive instruments and the limit. For liquid hazardous waste, as well as meeting the limits, it was necessary to get a new test method established.

Finally, the authors discussed what users expect from instrument manufacturers in addition to the XRF analyser. More and more users want a complete package with methods pre-loaded and calibration standards supplied. The next step may be to provide calibrated analysers 'ready to go'.

### Automated instrumentation for on-ship monitoring

**C. Leigh-Jones**
*Kittiwake Ltd, Littlehampton, UK*

Regular analysis of industrial fuel and lubricating oil is an integral part of routine machinery operation. Monitoring can be undertaken using a variety of techniques from in-line telemetry systems, to analysis in laboratories remote from the point of sampling. In 1990, the policy of the UK Royal Navy was changed from reliance upon shore based laboratories to analysis on board the vessels. In order to realize this change, it was necessary to improve the quality and accuracy of analysis equipment suitable for use in harsh industrial and marine environments.

The project was open to competitive tender and Kittiwake were chosen to undertake all design and development work. The first phase of the programme was to develop rugged and accurate equipment suitable for monitoring diesel engine crankcase lubricants. The equipment would be used to verify that the lubricant was 'fit for purpose' and suitable for continued use. Four parameters were identified as key indicators of this requirement, namely viscosity, base, number, insolubles and water contamination. Functional requirements written into the tender request were harsh and included performance criteria of:

- A maximum of 2 min for any one single test.
- Near laboratory accuracy (within 2% of laboratory test results).
- All equipment must be held in one single portable case.
- Equipment must pass a static drop test of 2.5 m.

The Kittiwake Oil Test Centre was developed and extensively tested before eventual fleet-wide implementation during 1996. It is estimated that the pay-back period for the Royal Navy on initial and outgoing investment will be within 12 months. The policy of on-board monitoring has now been adopted by three other Western navies with similar success. The current phase of development work is now nearing completion and will extend the application of existing equipment to cover gear box, turbine and hydraulic lubricants.

